# Supplementary material for: Metabolic shift toward ketosis in asocial cavefish increases social-like affinity
Source: BMC Biol. 2023 Oct 16;21:219. doi: 10.1186/s12915-023-01725-9 (PMC10577988; doi:10.1186/s12915-023-01725-9)
Supplement: Supplementary file 2 — Additional file 2: Figure S1. Two weeks of fasting reduced the glucose ketone index (GKI) in both surface fish and cavefish, and increased nearby interactions in cavefish. Figure S2. No detectable effect of the shift from live Artemia larvae (brine shrimp: BS) to the zebrafish standard control diet (CD) on the levels of serum glucose or ketone bodies, or nearby interactions. Figure S3. Brain ketone levels (beta-hydroxybutyrate) in cavefish under the CD or KD treatments. Figure S4. Ontogeny of swimming distance under ketogenic diet (KD) feeding. Figure S5. Consistent results were obtained in the repeated experiment for the duration and number of nearby interactions, and turning bias under control diet (CD) or ketogenic diet (KD) feeding. Figure S6. Daytime and nighttime number of sleeping events per hour under control diet (CD) or ketogenic diet (KD) feeding. Figure S7. Vibration attraction behavior (VAB) and swimming distance during VAB under control diet (CD) or ketogenic diet (KD) feeding. Figure S8. Five weeks of the ketone ester (KE)-supplemented diet feeding reduced glucose ketone index (GKI) in both surface fish and cavefish. Figure S9. Day and night sleeping durations, vibration attraction behavior (VAB) and growth were not drastically changed by ketone ester-supplemented diet (KE) feeding. Figure S10. Four weeks of the BHB salt-supplemented diet feeding reduced glucose ketone index (GKI) in both surface fish and cavefish. Figure S11. Nearby interactions and other behaviors under control diet (CD) or BHB sodium salt–supplemented diet (BHB) feeding. Figure S12. Day and night sleeping durations and vibration attraction behavior (VAB) were not drastically changed by beta-hydroxybutyrate-supplemented diet (BHB) feeding. Figure S13. No detectable effect of the hematocrit levels on serum glucose or ketone body. [file 12915_2023_1725_MOESM2_ESM.pdf]

Metabolic shift toward ketosis in asocial cavefish increases social-like affinity

Motoko Iwashita[1], Amity Tran[1], Marianne Garcia[1], Jia Cashon[1], Devanne Burbano[1], Vanessa Salgado[1], Malia Hasegawa[1], Rhoada Balmilero-Unciano[1], Kaylah Politan[1], Miki Wong[2,3], Ryan W.Y. Lee[4], and Masato Yoshizawa[1]*

[1] School of Life Sciences, the University of Hawai‘i at Mānoa, Honolulu, HI 96822, USA
[2] Nā Pu‘uwai Native Hawaiian Healthcare System, Kaunakakai, HI 96748, USA
[3] Nutrition Services Department, Shriners Hospitals for Children, Honolulu, HI 96826, USA
[4] Medical Staff Department, Shriners Hospitals for Children, Honolulu, HI 96826, USA

Corresponding author:
Masato Yoshizawa
yoshizaw@hawaii.edu

Additional File 2 : Figure S1-S13

Fig. S1: Two weeks of fasting reduced the glucose ketone index (GKI) in both surface fish and cavefish, and increased nearby interactions in cavefish.

Fig. S2: No detectable effect of the shift from live Artemia larvae (brine shrimp: BS) to the zebrafish standard control diet (CD) on the levels of serum glucose or ketone bodies, or nearby interactions.

Fig. S3: Brain ketone levels (beta-hydroxybutyrate) in cavefish under the CD or KD treatments.

Fig. S4: Ontogeny of swimming distance under ketogenic diet (KD) feeding.

Fig. S5: Consistent results were obtained in the repeated experiment for the duration and number of nearby interactions, and turning bias under control diet (CD) or ketogenic diet (KD) feeding.

Fig. S6: Daytime and nighttime number of sleeping events per hour under control diet (CD) or ketogenic diet (KD) feeding.

Fig. S7: Vibration attraction behavior (VAB) and swimming distance during VAB under control diet (CD) or ketogenic diet (KD) feeding.

Fig. S8: Five weeks of the ketone ester (KE)-supplemented diet feeding reduced glucose ketone index (GKI) in both surface fish and cavefish.

Fig. S9: Day and night sleeping durations, vibration attraction behavior (VAB) and growth were not drastically changed by ketone ester-supplemented diet (KE) feeding.

Fig. S10: Four weeks of the BHB salt-supplemented diet feeding reduced glucose ketone index (GKI) in both surface fish and cavefish.

Fig. S11: Nearby interactions and other behaviors under control diet (CD) or BHB sodium salt–supplemented diet (BHB) feeding.

Fig. S12: Day and night sleeping durations and vibration attraction behavior (VAB) were not drastically changed by beta-hydroxybutyrate-supplemented diet (BHB) feeding.

Fig. S13: No detectable effect of the hematocrit levels on serum glucose or ketone body

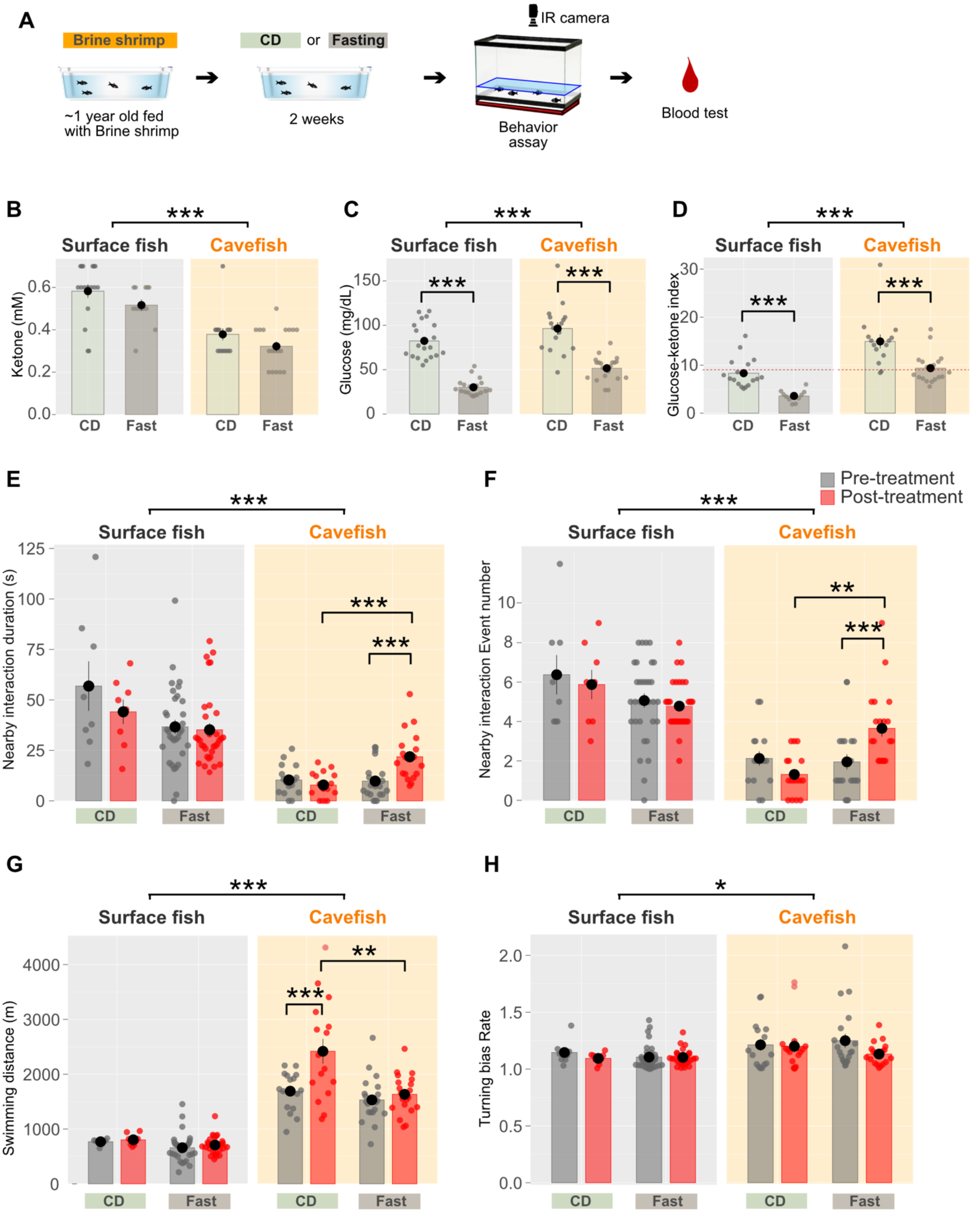

Fig. S1: Two weeks of fasting reduced the glucose ketone index (GKI) in both surface fish and cavefish, and increased nearby interactions in cavefish.

(A) Experimental procedure. Fish were either fasted (Fasting) or fed with brine shrimp (CD) for 2 weeks, after which blood glucose and ketone levels were measured. (B) Blood ketone level (mmol/L).

The 2-week fasting did not result in detectable changes in ketone levels in either surface fish (SF) or cavefish (CF). Data are presented as the mean ± standard error of the mean. Dots indicate individual data points. (C) Blood glucose level (mg/dL). Fasting significantly reduced glucose levels in both SF and CF. (D) The glucose ketone index (GKI) indicated that the ratio of glucose to ketone was lowered by fasting in both SF and CF, suggesting that this diet altered the balance between glucose and ketone. (E) Duration of nearby interactions in the 5-min assays. The time periods when a fish was within proximity (≤5 cm) and spent more than 4 s in a 5-min assay were summed for each fish. Fasted cavefish showed longer nearby interactions than the fed control. (F) Number of nearby interaction events. The number of nearby events (≤5 cm and ≥4 s) in a 5-min assay was counted. Fasted cavefish showed a higher number of nearby interactions than the fed control. (G) Swimming distance in a 5-min assay. Cavefish swam a longer distance than surface fish, and the fasted cavefish swam a shorter than the CD-fed cavefish after the 2 weeks treatment. (H) Turning biases of surface fish (left) and cavefish (right). Cavefish exhibited a higher turning index than surface fish, but none of the treatments induced further turning bias. See also Figure 4.
SF: N = 8 for CD feeding, N = 5 for fasting. CF: N = 8 for CD feeding, N = 8 for fasting. *: $P < 0.05$, **: $P < 0.01$, ***: $P < 0.001$. All detailed statistical data are available in Additional File 3.

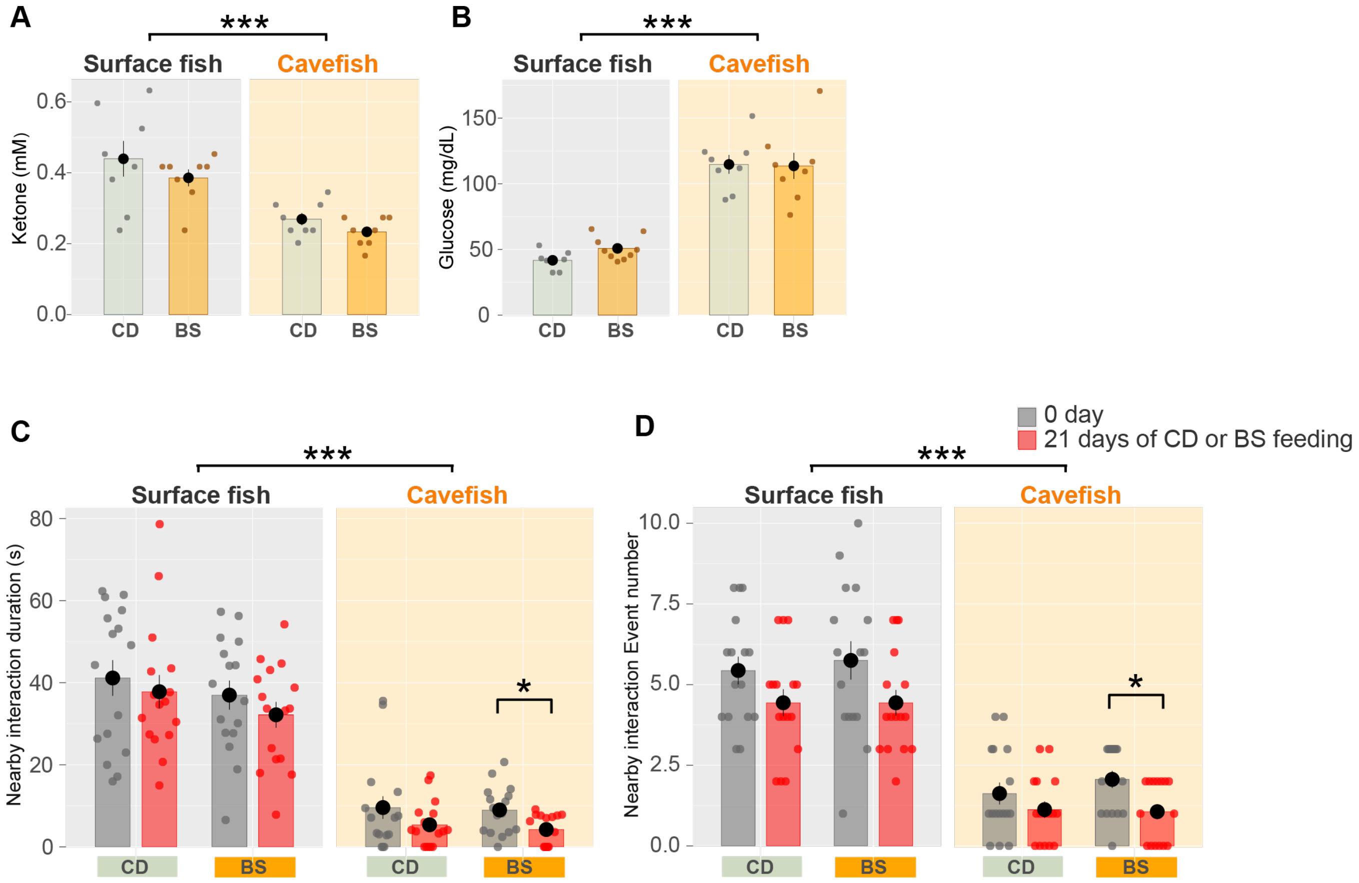

Fig. S2: No detectable effect of the shift from live Artemia larvae (brine shrimp: BS) to the zebrafish standard control diet (CD) on the levels of serum glucose or ketone bodies, or nearby interactions.

(A) Blood ketone level (mmol/L). After three weeks of treatment, ketone levels did not show detectable changes between CD and BS feeding in either surface fish or cavefish. Data are presented as the mean ± standard error of the mean. Dots indicate individual data points. (B) Blood glucose level (mg/dL). Glucose levels were not significantly changed by CD feeding in either surface or cavefish. (C) Duration of nearby interactions (s). During the 3 weeks of development, the average of the duration of nearby interactions was slightly reduced, but this was only detectable in BS-fed cavefish. (D) Number of nearby interaction events. The number of nearby interactions showed a similar result to (C): a slight reduction in interactions was detected in BS-fed cavefish. N = 20 for all groups. *: P < 0.05, **: P < 0.01, ***: P < 0.001. For (A) and (B), N = 8 for each group, For (C) and (D), N= 16 for each group. *: P < 0.05, **: P < 0.01, ***: P < 0.001.

All detailed statistical data are available in Additional File 3.

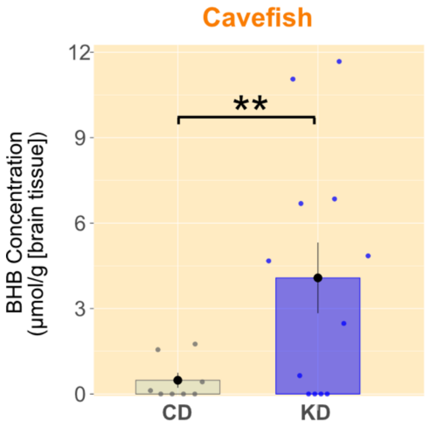

Fig. S3: Brain ketone levels (beta-hydroxybutyrate) in cavefish under the CD or KD treatments. The concentrations of beta-hydroxybutyrate in the cavefish brain tissues were measured in 8 individuals fed the control diet (CD) and 12 individuals fed the ketogenic diet (KD) after 1 month of the diet treatment. The concentration was adjusted by the brain weight (g). Generalized linear model (family = Gamma) resulted in $X^2(1) = 10.5$, $P = 0.0012$ **. N = 8 for the CD-fed cavefish, N = 12 for the KD-fed cavefish.
All detailed statistical data are available in Additional File 3.

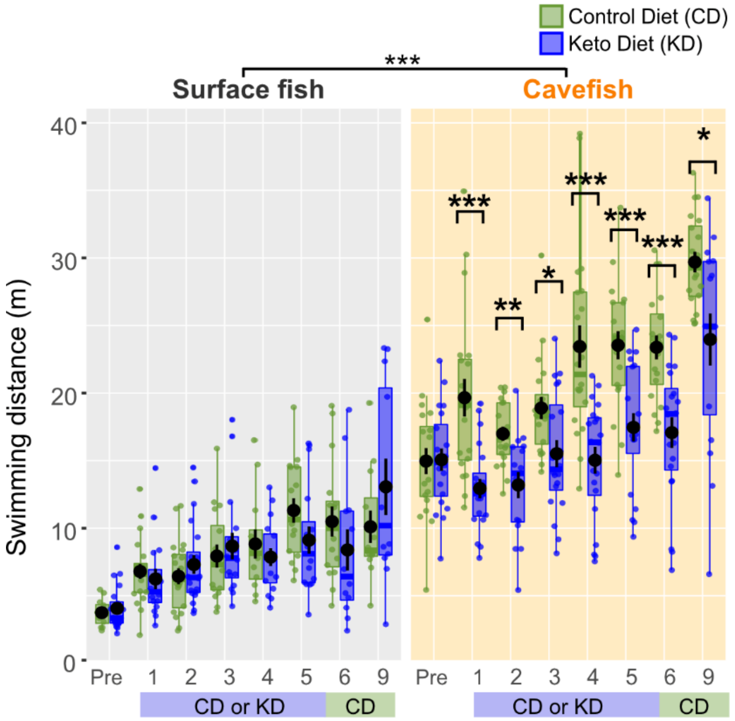

Fig. S4: Ontogeny of swimming distance under ketogenic diet (KD) feeding.
Surface fish exhibited an increase in the swimming distance over time under both the control diet (CD) and KD. In contrast, cavefish fed KD showed a suppression of the swimming distance and activity, starting in week 1, and these values remained smaller than those in CD-fed fish until week 9. However, KD-fed cavefish subsequently exhibited an increased swimming distance at week 9 after depriving KD feeding from week 6.
Data are presented as box plots. Dots indicate individual data. N = 20 in all groups. *: P < 0.05, **: P < 0.01, ***: P < 0.001. All detailed statistical data are available in Additional File 3.

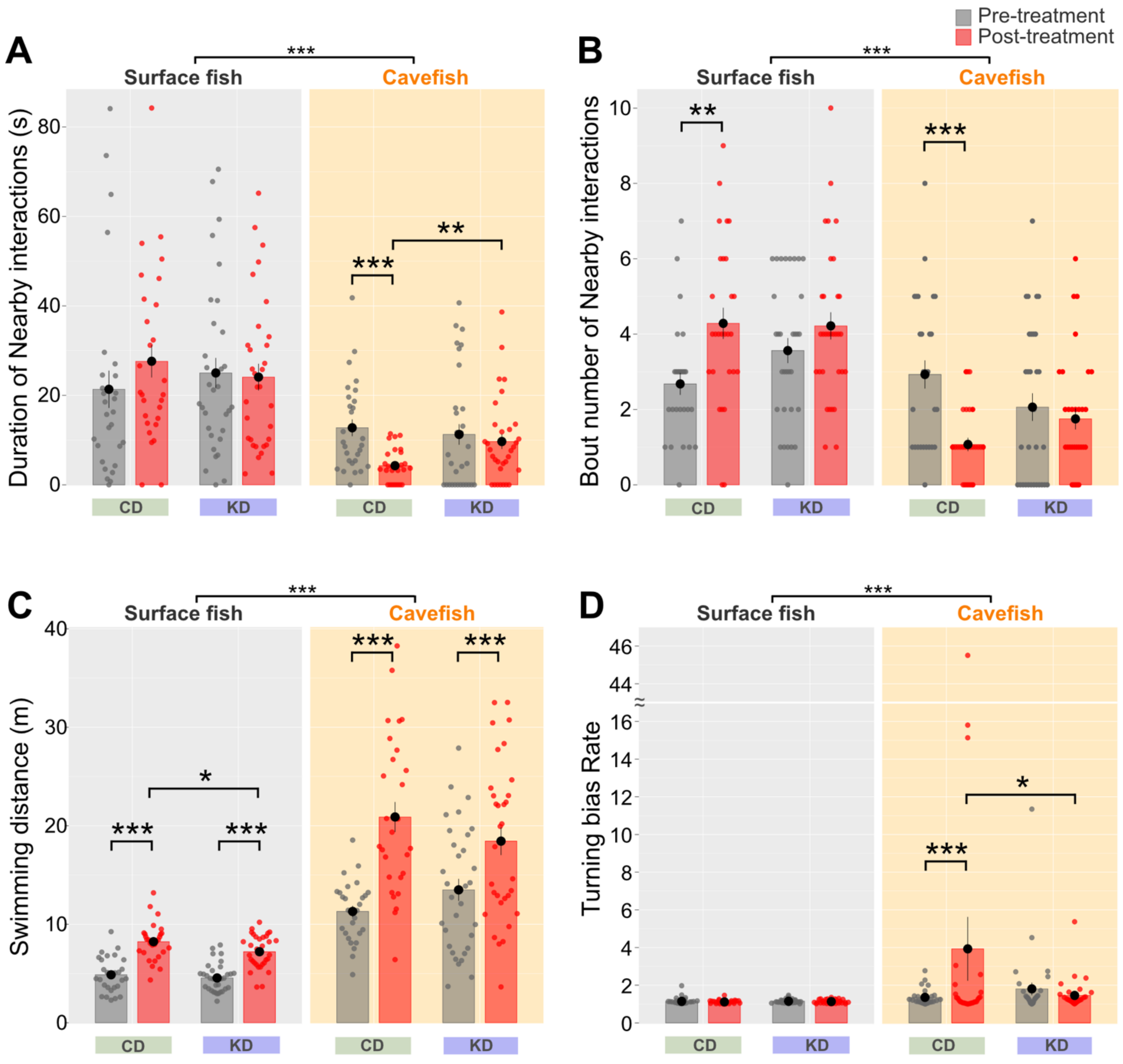

Fig. S5: Consistent results were obtained in the repeated experiment for the duration and number of nearby interactions, and turning bias under control diet (CD) or ketogenic diet (KD) feeding.

(A) Duration of nearby interactions, (B) Number of nearby interactions, (C) Swimming distance, and (D) Turning bias are presented. The overall tendencies were consistent with those observed in the original experiments, except for a shift in swimming distance (Figures 2 and 4). Surface fish did not exhibit any significant differences regarding the duration (A) or number of nearby interactions (B), the swimming distance (C), or turning bias (D). The duration (A) and number of nearby interactions (B) were maintained in KD-fed cavefish, while they were reduced in CD-fed cavefish, which also exhibited a higher level of turning bias (D). The swimming distance was not significantly reduced in KD-fed cavefish compared to CD-fed controls in this repeated experiment (C).

Data are presented as the mean ± standard error of the mean. Dots indicate individual data. Surface fish: N = 28 for CD, N = 32 for KD. Cavefish: N = 28 for CD, N = 32 for KD. *: $P < 0.05$, **: $P < 0.01$, ***: $P < 0.001$. All detailed statistical data are available in Additional File 3.

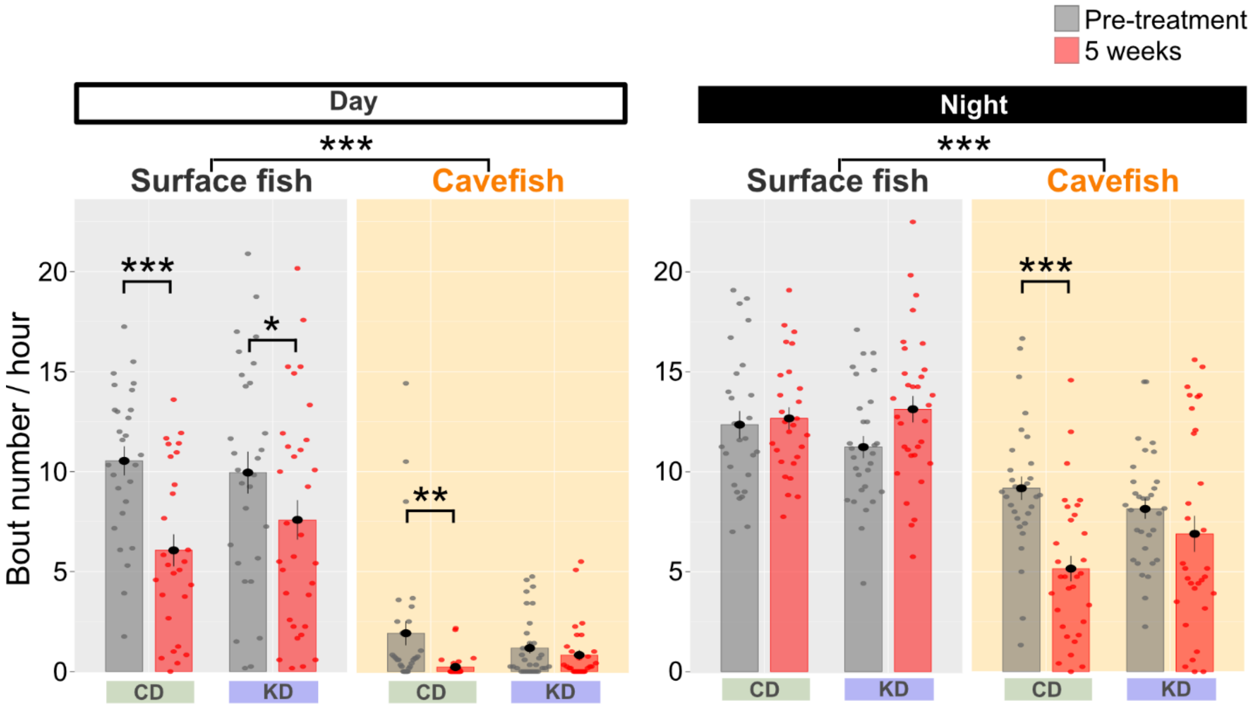

Fig. S6: Daytime and nighttime number of sleeping events per hour under control diet (CD) or ketogenic diet (KD) feeding.
After 5 weeks of growth, surface fish exhibited a reduced number of sleeping events during the day under both diets. CD-fed cavefish also exhibited a reduced number of sleeping events during the day and night. However, the number of sleeping events did not differ according to the diet in cavefish or surface fish.
Data are presented as the mean ± standard error of the mean. Dots indicate individual data points. Surface fish: N = 28 for CD, N = 32 for KD. Cavefish: N = 28 for CD, N = 32 for KD. *: $P < 0.05$, **: $P < 0.01$, ***: $P < 0.001$. All detailed statistical data are available in Additional File 3.

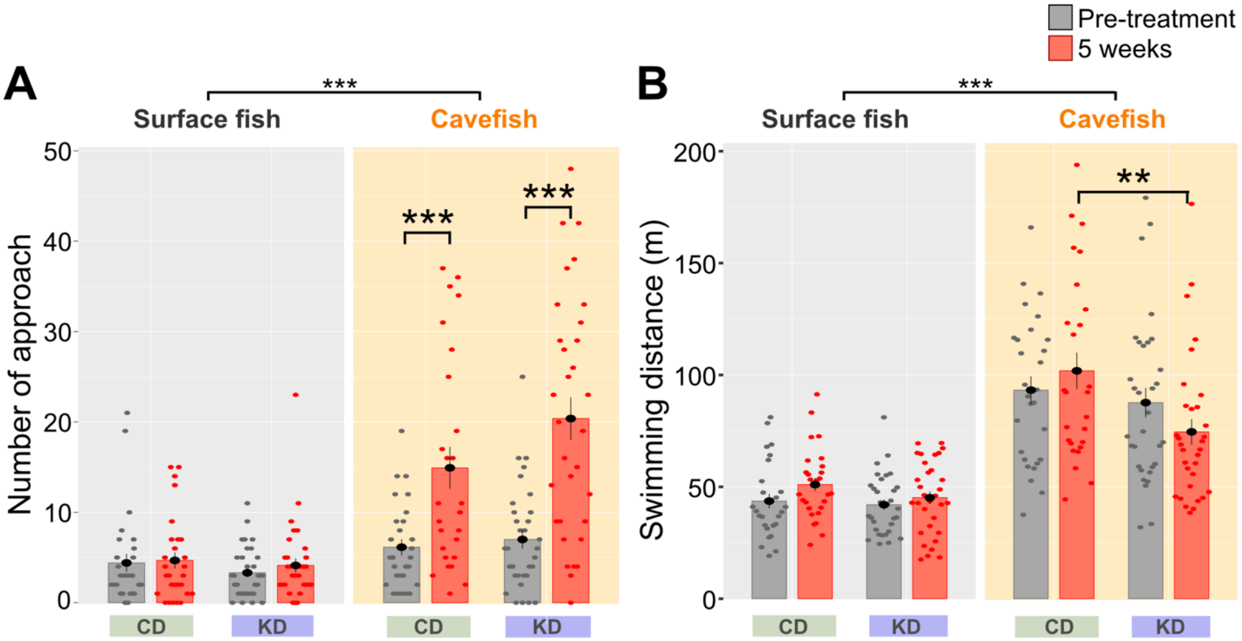

Fig. S7: Vibration attraction behavior (VAB) and swimming distance during VAB under control diet (CD) or ketogenic diet (KD) feeding.

(A) Number of approaches to the vibration rod in the 3-min assays. After 5 weeks of growth, the number of approaches increased in CD- and KD-fed cavefish, but no difference according to the diet was detected in either surface fish or cavefish. (B) Swimming distance during VAB. KD-fed cavefish swam significantly shorter distances than CD-fed cavefish, consistent with the nearby interaction and sleep studies.

Data are presented as the mean ± standard error of the mean. Dots indicate individual data points. Surface fish: N = 28 for CD, N = 32 for KD. Cavefish: N = 28 for CD, N = 32 for KD. *: $P < 0.05$, **: $P < 0.01$, ***: $P < 0.001$. All detailed statistical data are available in Additional File 3.

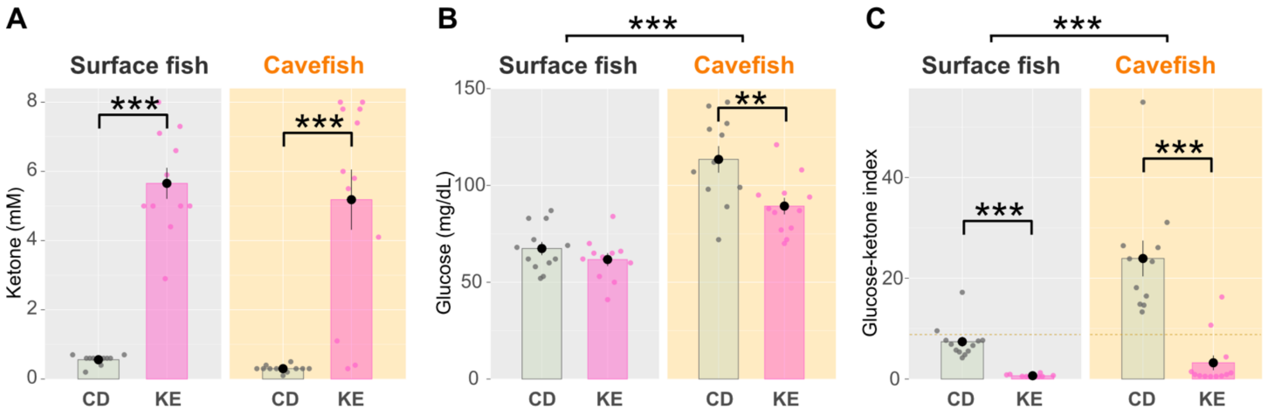

Fig. S8: Five weeks of the ketone ester (KE)-supplemented diet feeding reduced glucose ketone index (GKI) in both surface fish and cavefish.
(A) Blood ketone level (mmol/L). Ketone levels significantly changed after the 5-week feeding in both surface fish and cavefish. Data are presented as the mean ± standard error of the mean. Dots indicate individual data points. (B) Blood glucose level (mg/dL). Glucose levels were significantly reduced in cavefish (CF). (C) The glucose ketone index (GKI) indicated that the ratio of glucose to ketone was lowered by the 5-week KE supplemental feeding in both surface fish (SF) and CF, suggesting that this diet altered the balance between glucose and ketone.
SF: N = 12 for CD feeding, N = 10 for KE feeding. CF: N = 11 for CD feeding, N = 12 for KE feeding. *: $P < 0.05$, **: $P < 0.01$, ***: $P < 0.001$. All detailed statistical data are available in Additional File 3.

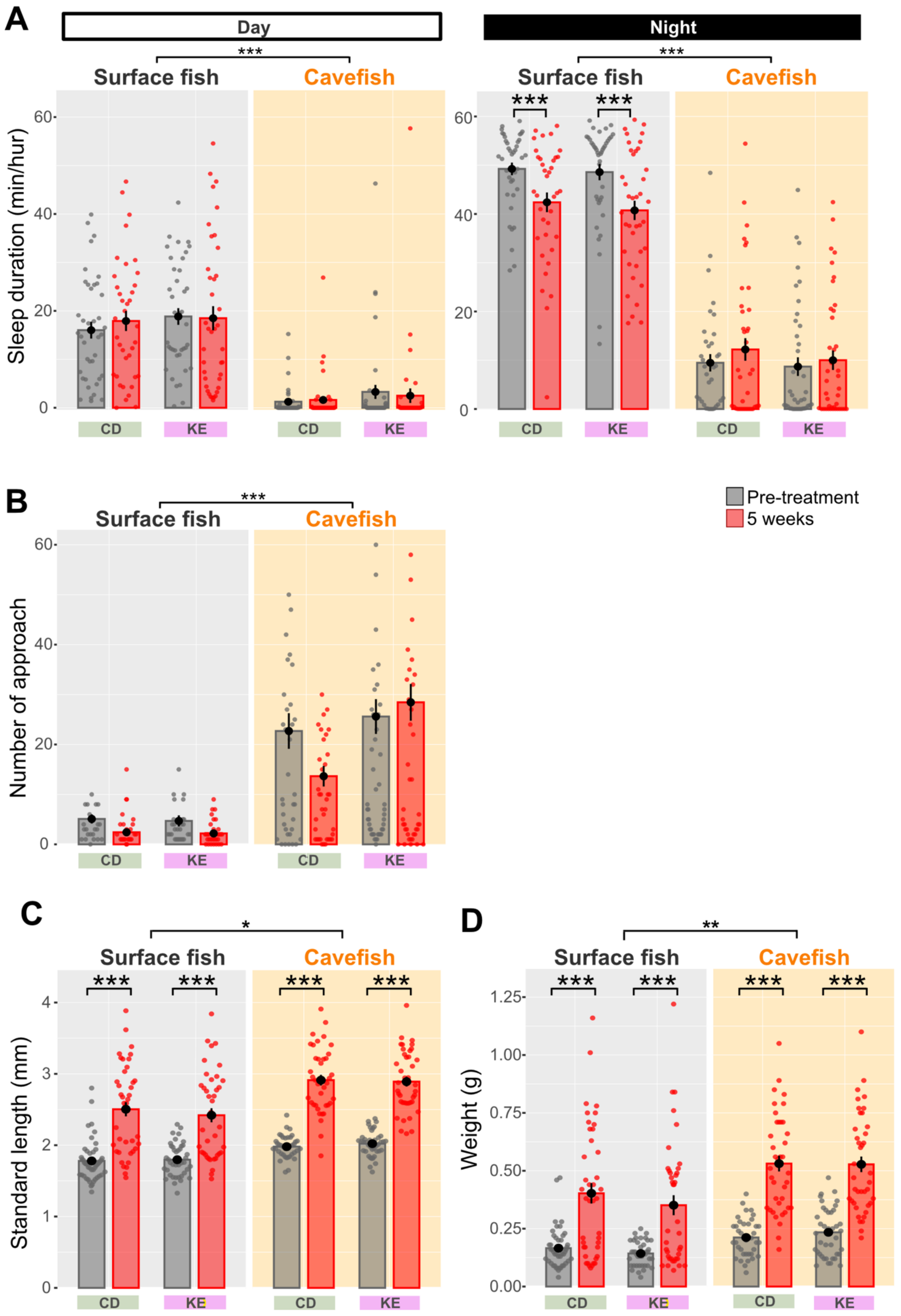

Fig. S9: Day and night sleeping durations, vibration attraction behavior (VAB) and growth were not drastically changed by ketone ester-supplemented diet (KE) feeding.

(A) Sleep duration (min/h) during the day (left) and night (right). During the 5 weeks of treatment, neither surface fish nor cavefish exhibited any detectable changes in KE feeding, although the night-time sleep in surface fish was reduced after 5 weeks of growth. (B) Number of approaches per the 3-min assay (VAB level). During the 5 weeks of treatment, the VAB level did not detectably shift in surface fish or cavefish regardless of diets. (C) Standard length (cm). KE-fed surface fish and cavefish were comparable to their CD-fed counterparts. (D) Body weight (g). KE-fed surface fish and cavefish weighed comparable to their CD-fed counterparts.
Data are presented as the mean ± standard error of the mean. Dots indicate individual data points. For (A) and (B), Surface fish: N = 37 for CD, N = 39 for KE. Cavefish: N = 39 for CD, N = 40 for KE. For (C) and (D), Surface fish: N = 40 for CD, N = 39 for KE. Cavefish: N = 39 for CD, N = 40 for KE. *: $P < 0.05$, **: $P < 0.01$, ***: $P < 0.001$. All detailed statistical data are available in Additional File 3.

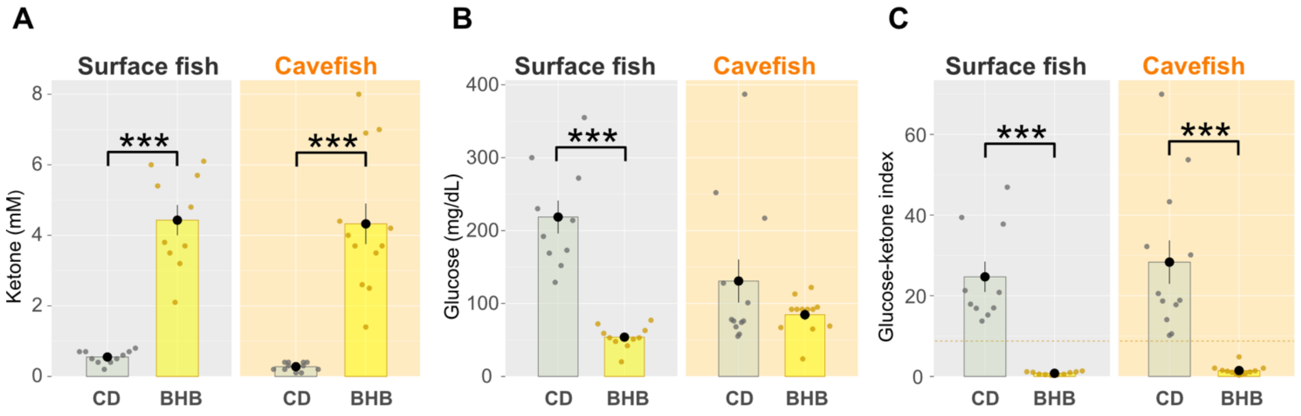

Fig. S10: Four weeks of the BHB salt-supplemented diet feeding reduced glucose ketone index (GKI) in both surface fish and cavefish.

(A) Blood ketone level (mmol/L). Ketone levels significantly changed by the 4-week feeding in both surface fish (SF) and cavefish (CF). Data are presented as the mean ± standard error of the mean. Dots indicate individual data. (B) Blood glucose level (mg/dL). Glucose levels were significantly reduced in SF. (C) The glucose ketone index (GKI) indicated that the ratio of glucose to ketone was lowered by the 4-week BHB salt-supplemented feeding in both SF and CF, suggesting that this diet altered the balance between glucose and ketone.

SF: N = 10 for CD feeding, N = 10 for BHB feeding. CF: N = 10 for CD feeding, N = 10 for BHB feeding. *: $P < 0.05$, **: $P < 0.01$, ***: $P < 0.001$. All detailed statistical data are available in Additional File 3.

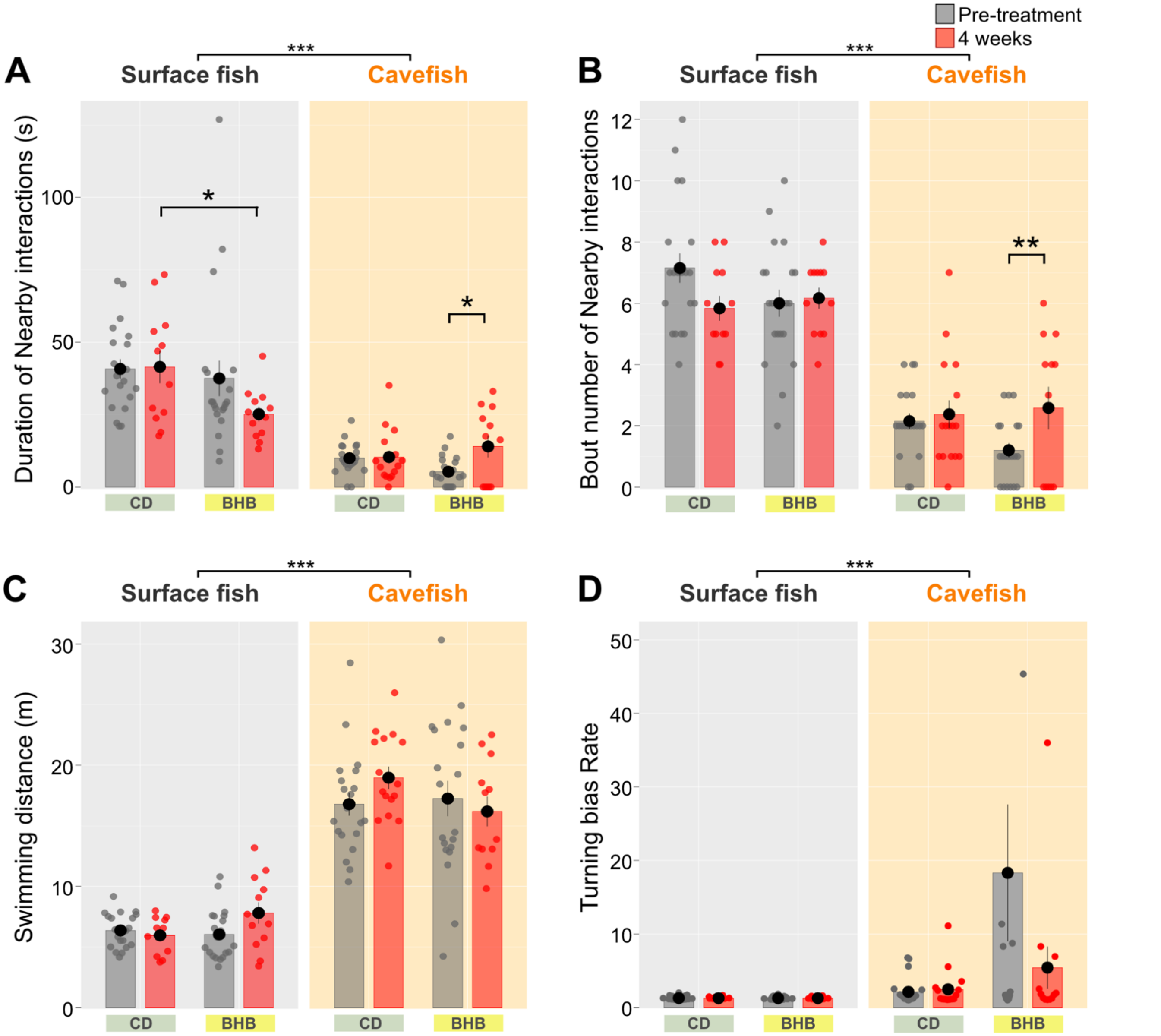

Fig. S11: Nearby interactions and other behaviors under control diet (CD) or BHB sodium salt–supplemented diet (BHB) feeding.
(A) Duration of nearby interactions (s). After 4 weeks, the duration of nearby interactions decreased in BHB-treated surface fish and increased in BHB-treated cavefish. (B) Number of nearby interactions. The number of nearby interactions increased in BHB-treated cavefish. (C) Swimming distance. No difference was detected between the CD and BHB groups. (D) Turning bias ratio. BHB-treated cavefish tended to exhibit decreased biased turning, although this reduction was not significant.
Data are presented as the mean ± standard error of the mean. Dots indicate individual data points. N = 20 for all groups. *: $P < 0.05$, **: $P < 0.01$, ***: $P < 0.001$. All detailed statistical data are available in Additional File 3.

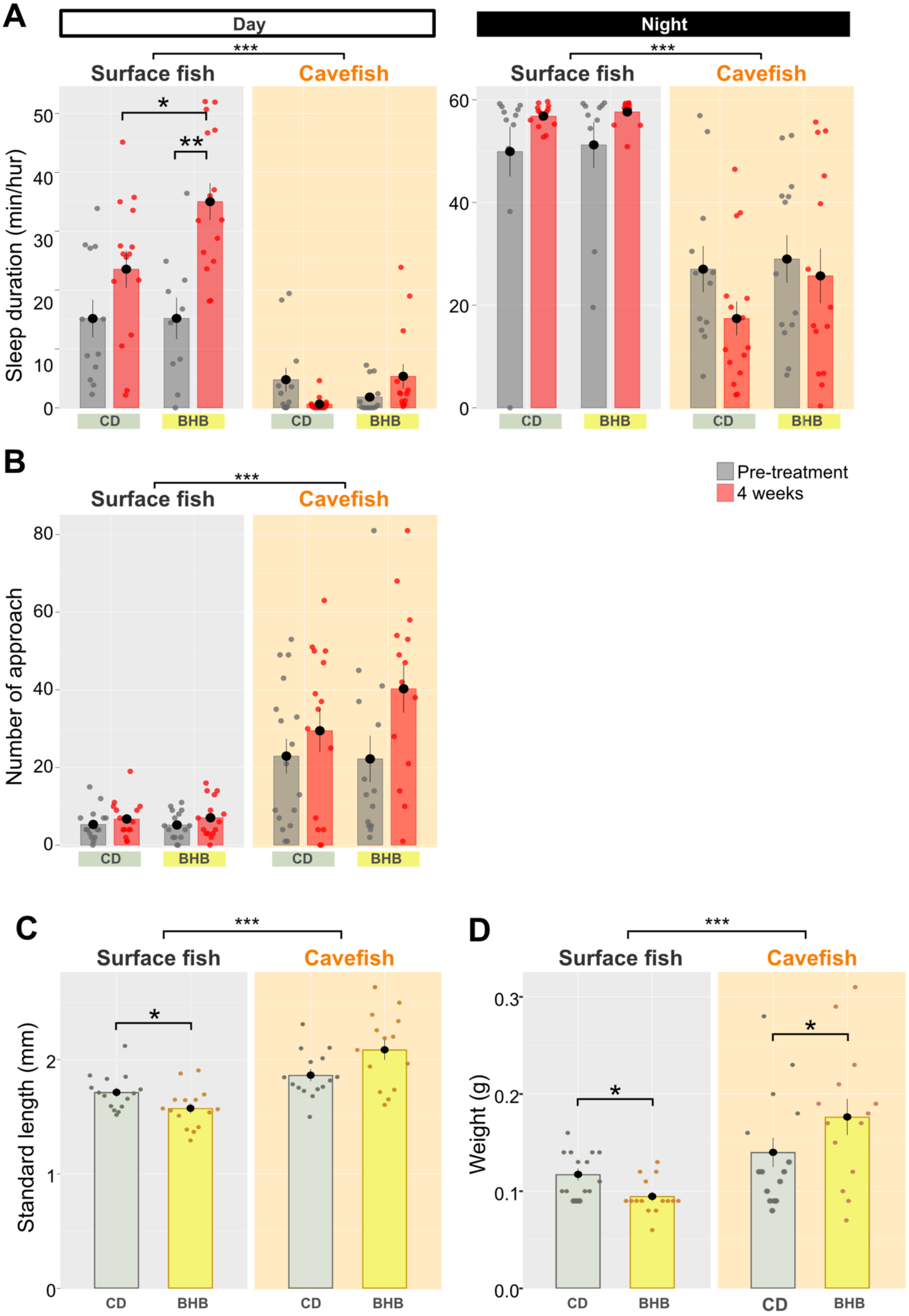

Fig. S12: Day and night sleeping durations and vibration attraction behavior (VAB) were not drastically changed by beta-hydroxybutyrate-supplemented diet (BHB) feeding.

(A) Sleep duration (min/h) during the day (left) and night (right). During 4 weeks of treatment, the daytime sleep duration increased in surface fish under BHB feeding but not during nighttime. Cavefish did not exhibit any detectable changes. (B) Number of approaches per the 3-min assay (VAB level). During 4 weeks of treatment, the VAB level did not shift in surface fish or cavefish regardless of the diets. Data are presented as the mean ± standard error of the mean. Dots indicate individual data points. N = 20 for all groups. (C) Standard length (cm). The BHB-treated surface fish grew slower than the control diet-treated ones. Cavefish showed no detectable change between the control and BHB supplementation. (D) Body weight (g). BHB-treated surface fish exhibited significantly reduced weight, whereas the weight of BHB-fed cavefish did not show any negative effect on the growth.
Data are presented as the mean ± standard error of the mean. Dots indicate individual data. N = 20 for all groups. *: $P < 0.05$, **: $P < 0.01$, ***: $P < 0.001$. All detailed statistical data are available in Additional File 3.

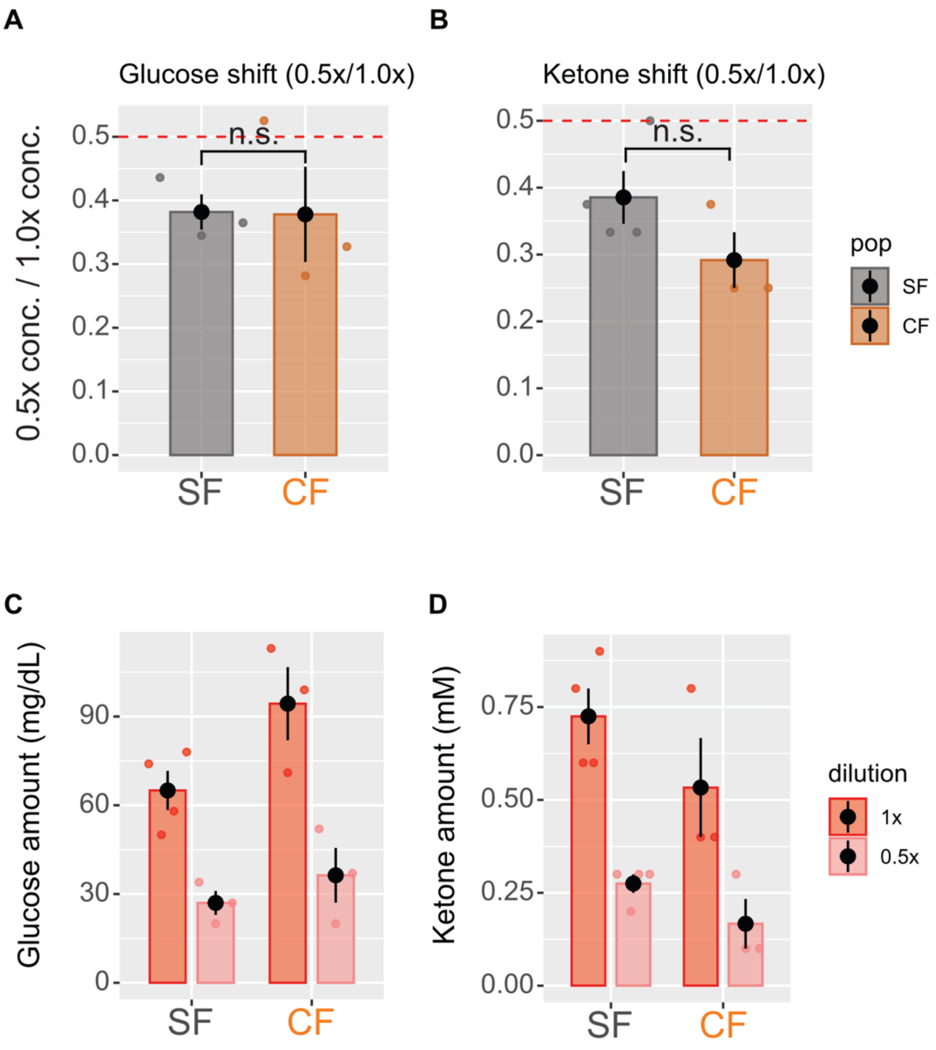

Fig. S13: No detectable effect of the hematocrit levels on serum glucose or ketone body measurements. The blood of three surface fish and three cavefish was individually collected. Each blood sample was divided into non-diluted (1.0x) and diluted (0.5x) portions diluted with phosphate buffered saline (PBS). The blood samples were then measured using by Abbott Precision Xtra. If the difference of the hematocrit levels between surface and cavefish (28.51 ± 0.03% and 35.56± 0.03%, respectively) affected the readings of glucose or ketone bodies, the diluted/undiluted ratios would show a significant difference between surface fish and cavefish. (A) Ratio of glucose concentration in diluted (0.5x) and non-diluted (1.0x) blood. The expected ratio is 0.5, but it was shifted towards a lower concentration, possibly due to the low hematocrit level (~15%) in 0.5x. However, the ratio of glucose concentrations showed no detectable difference between surface fish and cavefish, suggesting that the different hematocrit levels did not have a significant effect on the serum glucose readings. (B) Ratio of ketone bodies concentration in diluted (0.5x) and non-diluted (1.0x) blood. Similar to (A), the ratio showed no significant difference between surface fish and cavefish, indicating that the different hematocrit levels between the two populations had little effect on the ketone body concentration readings. (C) Raw measurements of the glucose concentrations used in (A). (D) Raw measurements of the ketone body concentrations used in (B). Detailed statistical scores are available in Additional File 3.
