# Supplementary material for: Metabolic shift toward ketosis in asocial cavefish increases social-like affinity
Source: BMC Biol. 2023 Oct 16;21:219. doi: 10.1186/s12915-023-01725-9 (PMC10577988; doi:10.1186/s12915-023-01725-9)
Supplement: Supplementary file 4 — Additional file 4. Table of possible biological processes in each behavior tested in this study. [file 12915_2023_1725_MOESM4_ESM.pdf]

Additional file 4. Possible biological processes in each behavior tested in this study.

Behavior	Results in this study	Known biological pathway or processes
Social behavior [1, 2]	Promoted	Dopaminergic,<br>Serotonergic,<br>Oxytocinergic,<br>Learning and memory processes
Repetitive behavior [3]	Promoted	Dopaminergic<br>Serotonergic<br>GABAergic<br>Glutamatergic<br>Synaptic plasticity processes
Vibration attraction behavior (foraging, and adherence) [4–6]	No detectable change	Orexinergic (?),<br>Ghrelin (?),<br>Peptide Y (?),<br>cholecystokinin (?)
Sleep [7–10]	No detectable change	Histaminergic,<br>Cholinergic,<br>Serotonergic,<br>Orexinergic/Hypocretinergic<br>Glutamatergic,<br>Glycinergic,<br>GABAergic<br>Noradrenergic

(?): implied by assuming VAB as a foraging behavior. Underlined pathways appeared in GO term/KEGG analysis too (see in the Discussion).

References:

1. Churchland PS, Winkielman P. Modulating social behavior with oxytocin: How does it work? What does it mean? Hormones and Behavior. 2012;61:392–9.

2. Kiser D, SteemerS B, Branchi I, Homberg JR. The reciprocal interaction between serotonin and social behaviour. Neuroscience and Biobehavioral Reviews. 2012;36:786–98.

3. Langen M, Kas MJH, Staal WG, van Engeland H, Durston S. The neurobiology of repetitive behavior: Of mice... Neuroscience and Biobehavioral Reviews. 2011;35:345–55.

4. Penney CC, Volkoff H. Peripheral injections of cholecystokinin, apelin, ghrelin and orexin in cavefish (Astyanax fasciatus mexicanus): Effects on feeding and on the brain expression levels of tyrosine hydroxylase, mechanistic target of rapamycin and appetite-related hormones. Gen Comp Endocrinol. 2014;196:34–40.

5. Wall A, Volkoff H. Effects of fasting and feeding on the brain mRNA expressions of orexin, tyrosine hydroxylase (TH), PYY and CCK in the Mexican blind cavefish (*Astyanax fasciatus mexicanus*). General and Comparative Endocrinology. 2013;183:44–52.

6. Barson JR. Orexin/hypocretin and dysregulated eating: Promotion of foraging behavior. Brain Research. 2020;1731 May 2018:145915.

7. Jaggard JB, Stahl BA, Lloyd E, Duboue ER, Keene AC, Prober DA, et al. Hypocretin underlies the evolution of sleep loss in the Mexican cavefish. eLife. 2017;7.

8. Jaggard JB, Lloyd E, Yuiska A, Patch A, Fily Y, Kowalko JE, et al. Cavefish brain atlases reveal functional and anatomical convergence across independently evolved populations. Science advances. 2020;6:3126–42.

9. Siegel JM. The neurotransmitters of sleep. Journal of Clinical Psychiatry. 2004;65 SUPPL. 16:4–7.

10. Duboué ER, Keene AC, Borowsky RL. Evolutionary convergence on sleep loss in cavefish populations. Current Biology. 2011;21:671–6.
