# Supplementary material for: Metabolic shift toward ketosis in asocial cavefish increases social-like affinity
Source: BMC Biol. 2023 Oct 16;21:219. doi: 10.1186/s12915-023-01725-9 (PMC10577988; doi:10.1186/s12915-023-01725-9)

Molecular pathway analysis (using KEGG DB)

Data source: ASD risk genes having same expression pattern to that from patient (1,800 genes)

(Yoshizawa et. al., 2018)

Red: Nervous system; **Blue**: Metabolic system

hsa04721:Synaptic vesicle cycle	hsa04611:Platelet activation	hsa04144:Endocytosis
hsa04730:Long-term depression	hsa05230:Central carbon metabolism in cancer	hsa04024:cAMP signaling pathway
hsa04010:MAPK signaling pathway	hsa00330:Arginine and proline metabolism	hsa04918:Thyroid hormone synthesis
hsa04022:cGMP-PKG signaling pathway	hsa05169:Epstein-Barr virus infection	hsa01130:Biosynthesis of antibiotics
hsa05205:Proteoglycans in cancer	hsa04924:Renin secretion	hsa04966:Collecting duct acid secretion
hsa04070:Phosphatidylinositol signaling system	hsa04510:Focal adhesion	hsa04145:Phagosome
hsa04921:Oxytocin signaling pathway	hsa01230:Biosynthesis of amino acids	hsa05212:Pancreatic cancer
hsa04713:Circadian entrainment	hsa05142:Chagas disease (American trypanosomiasis)	hsa04720:Long-term potentiation
hsa00562:Inositol phosphate metabolism	hsa04066:HIF-1 signaling pathway	hsa00250:Alanine, aspartate and glutamate metabolism
hsa04261:Adrenergic signaling in cardiomyocytes	hsa04540:Gap junction	hsa05130:Pathogenic Escherichia coli infection
hsa04728:Dopaminergic synapse	hsa01200:Carbon metabolism	hsa04360:Axon guidance
hsa03050:Proteasome	hsa04971:Gastric acid secretion	hsa04972:Pancreatic secretion
hsa04970:Salivary secretion	hsa05120:Epithelial cell signaling in Helicobacter pylori infection	hsa05110:Vibrio cholerae infection
hsa04723:Retrograde endocannabinoid signaling	hsa00010:Glycolysis / Gluconeogenesis	hsa04015:Rap1 signaling pathway
hsa04071:Sphingolipid signaling pathway	hsa04915:Estrogen signaling pathway	hsa04726:Serotonergic synapse
hsa04724:Glutamatergic synapse	hsa04270:Vascular smooth muscle contraction	
hsa 04020:Calcium signaling pathway	hsa04141:Protein processing in endoplasmic reticulum	

Following slides: Each KEGG pathway related to the nervous system (red in the former slide) and metabolic system (blue in the former slide) is shown.

Star marks (★) in the following slides indicate the ASD risk genes that show shared directional gene expression patterns (up- or down-regulations compared with the typical individuals) between cavefish and patients with ASD.

Synaptic vesicle cycle

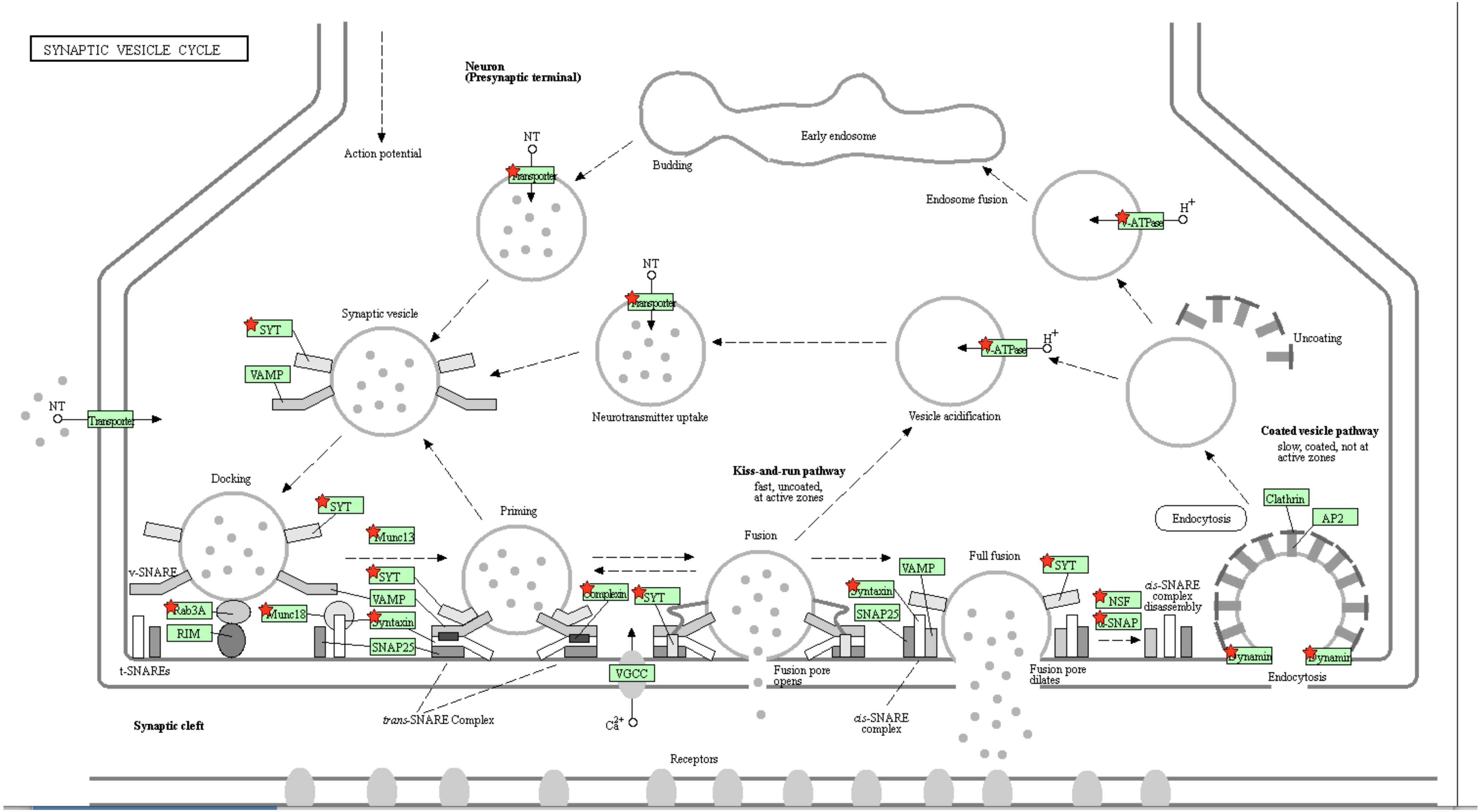

Long-term depression

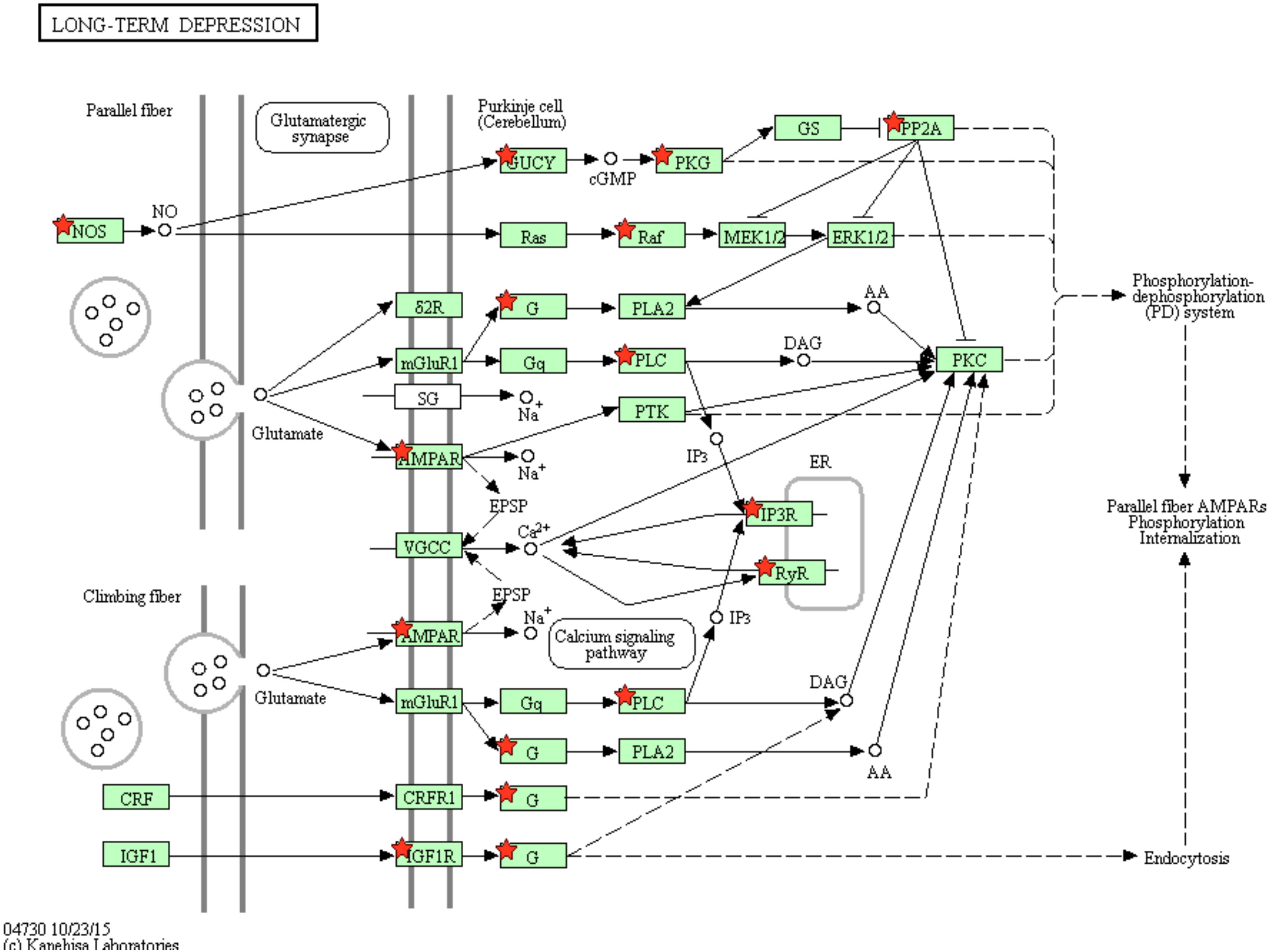

Oxytocin signaling pathway

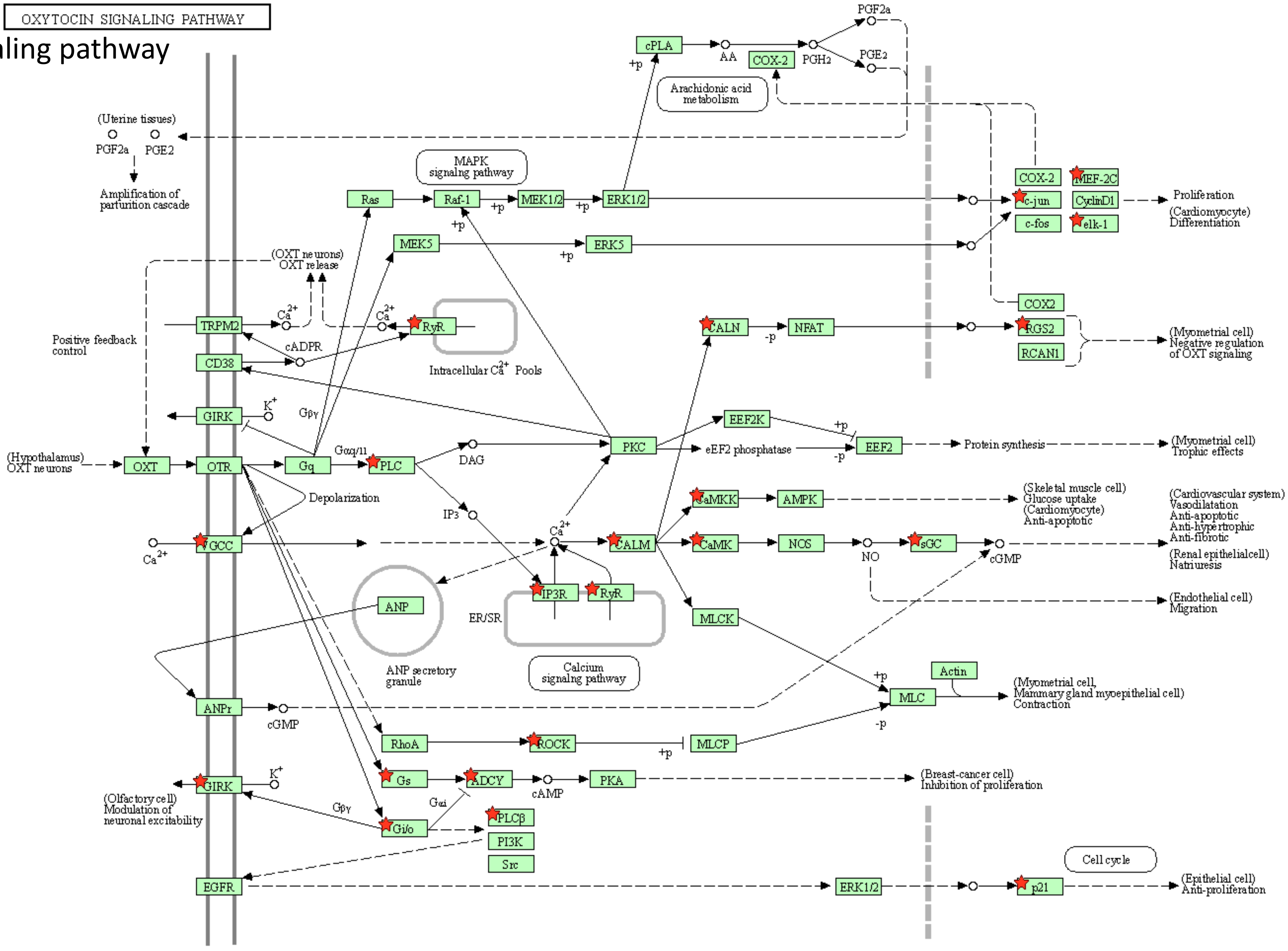

Dopaminergic synapse

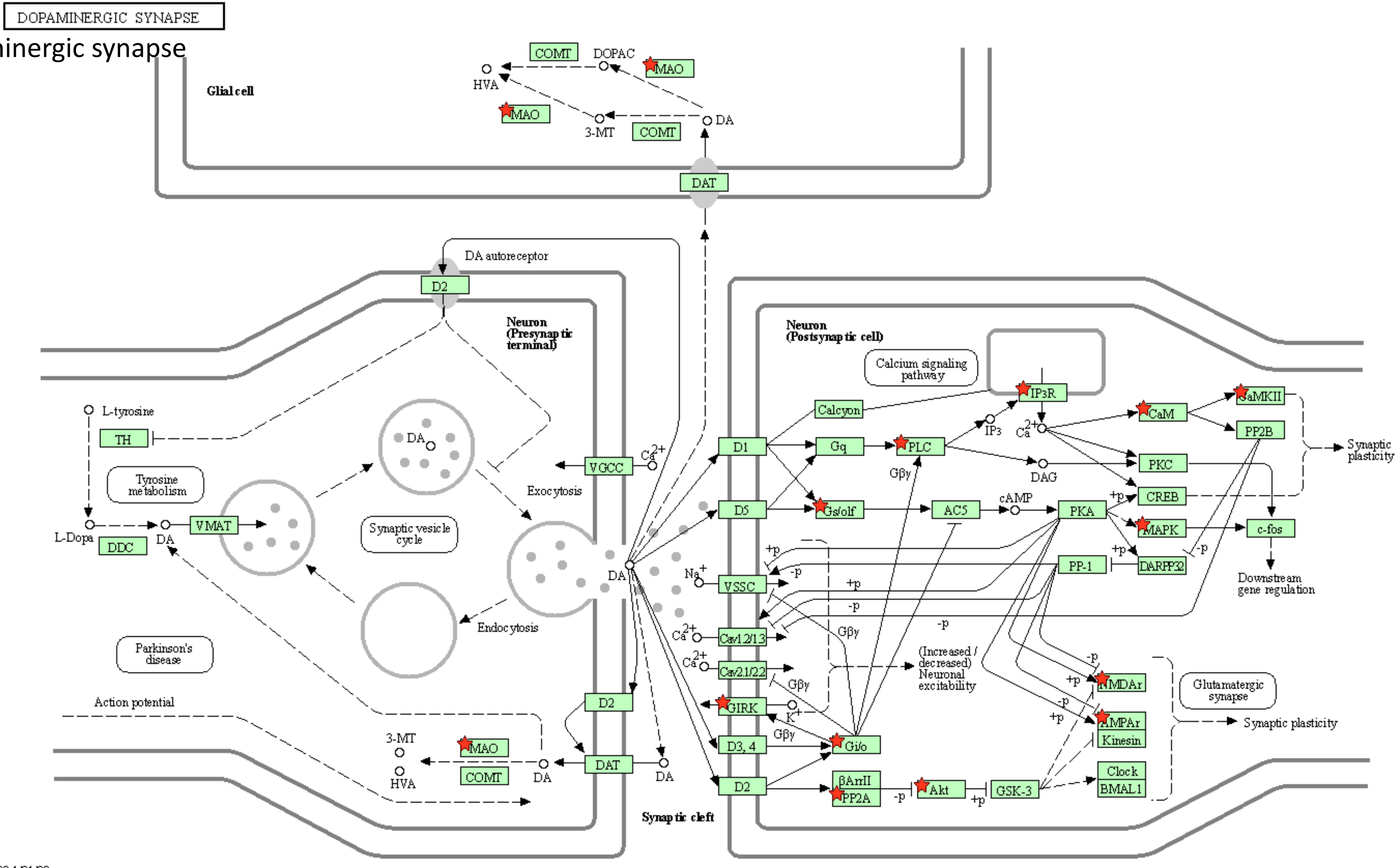

Glutamatergic synapse

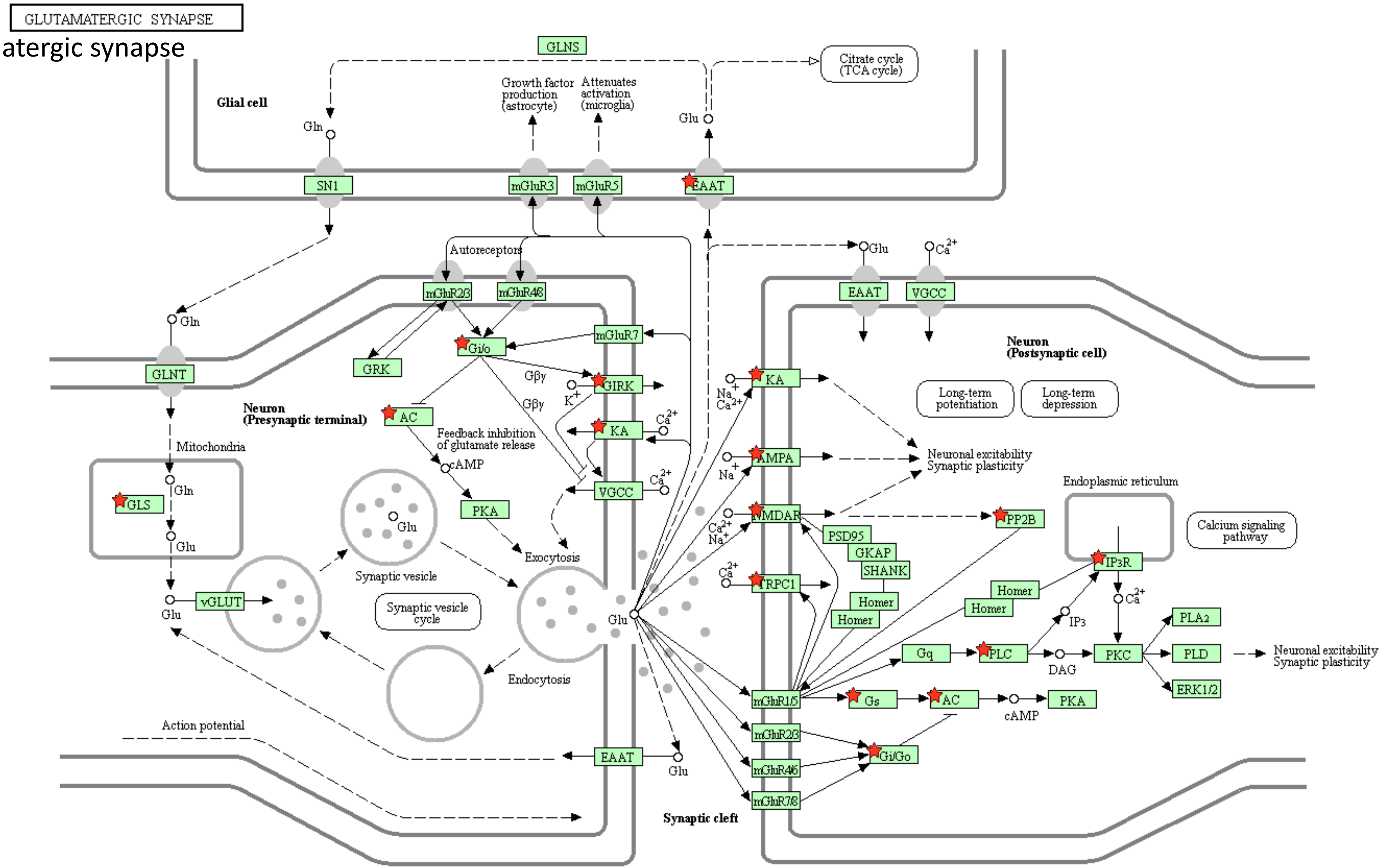

Central carbon metabolism in cancer

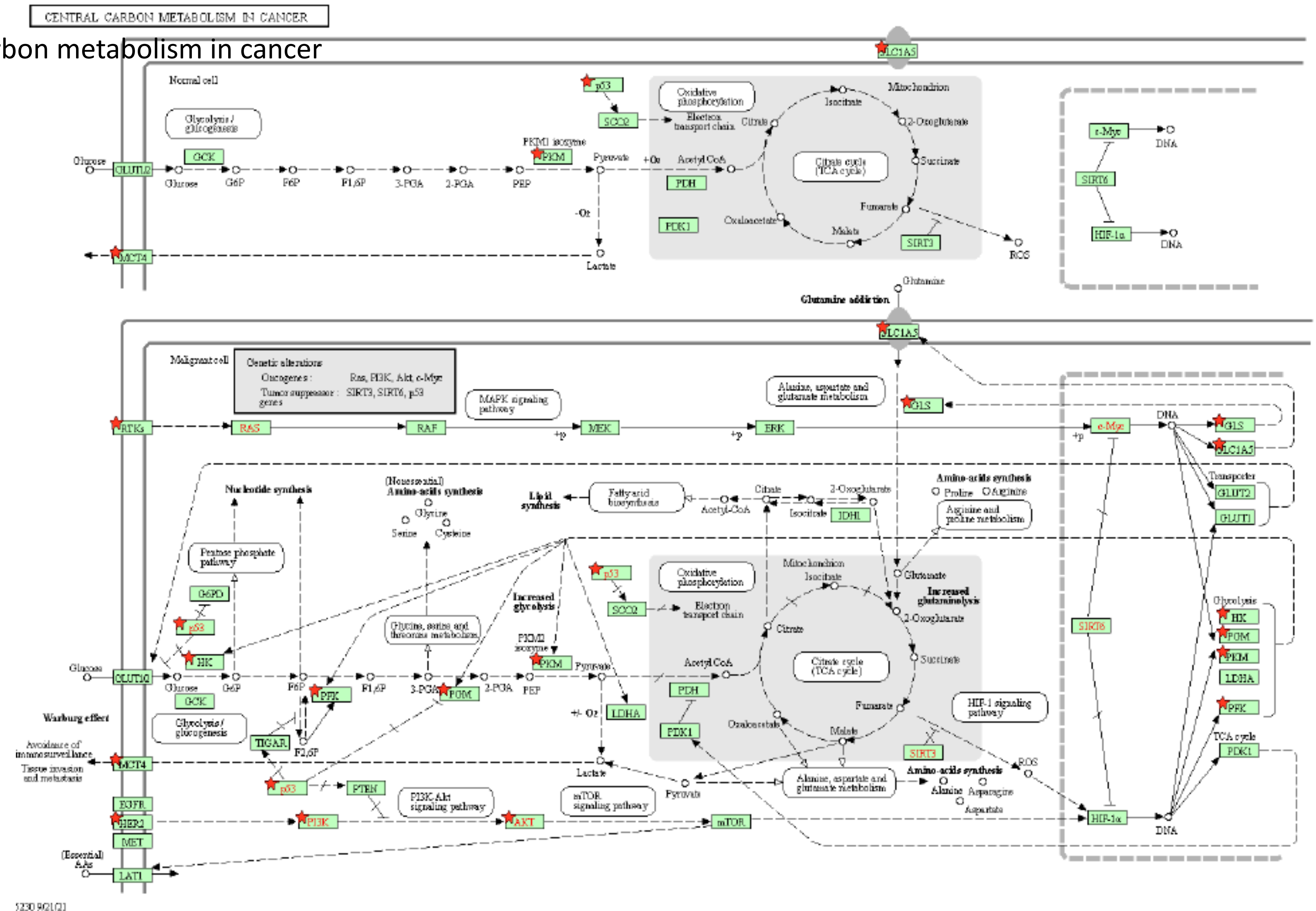

Glycolysis / Gluconeogenesis

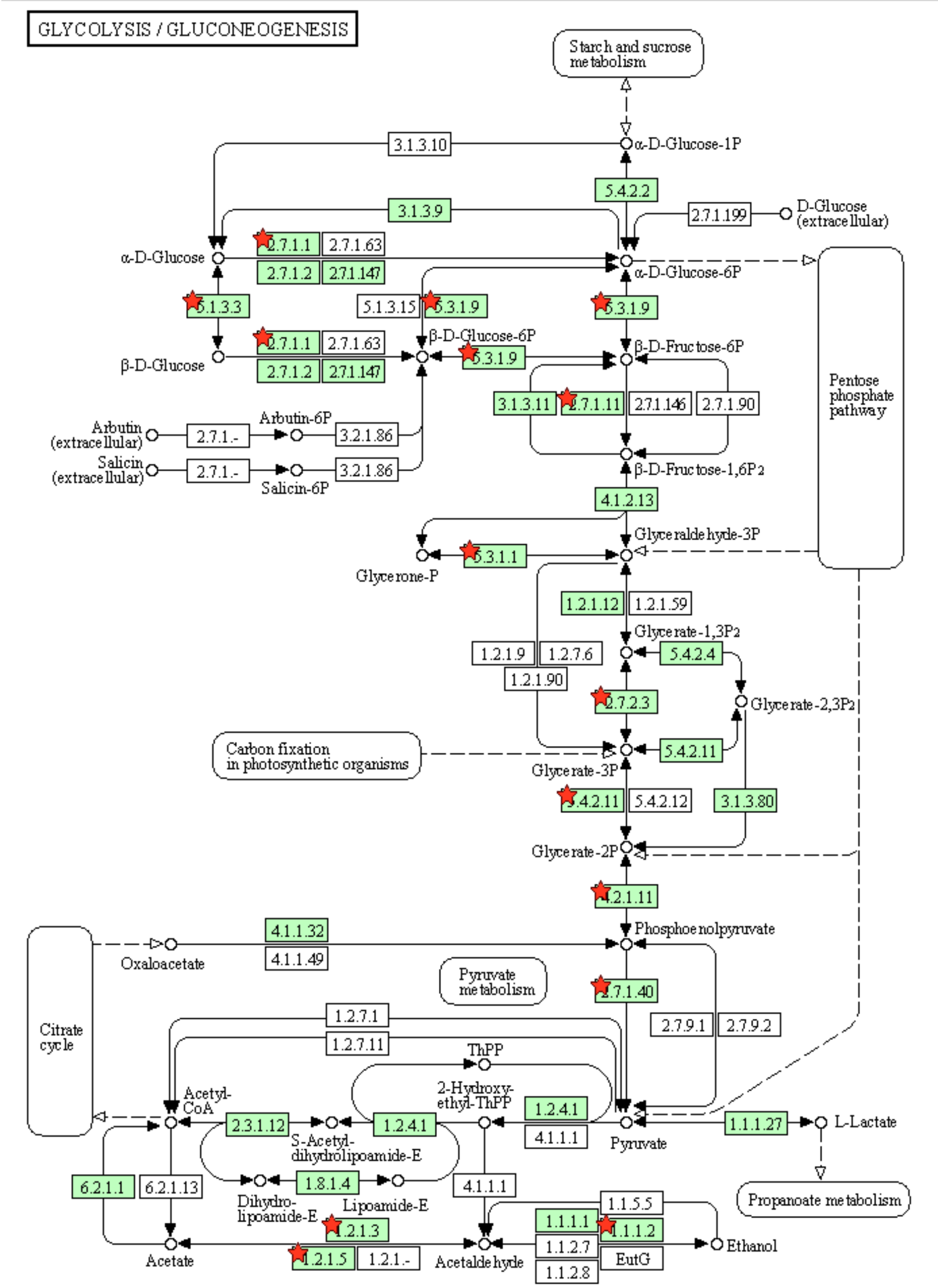

Endocytosis

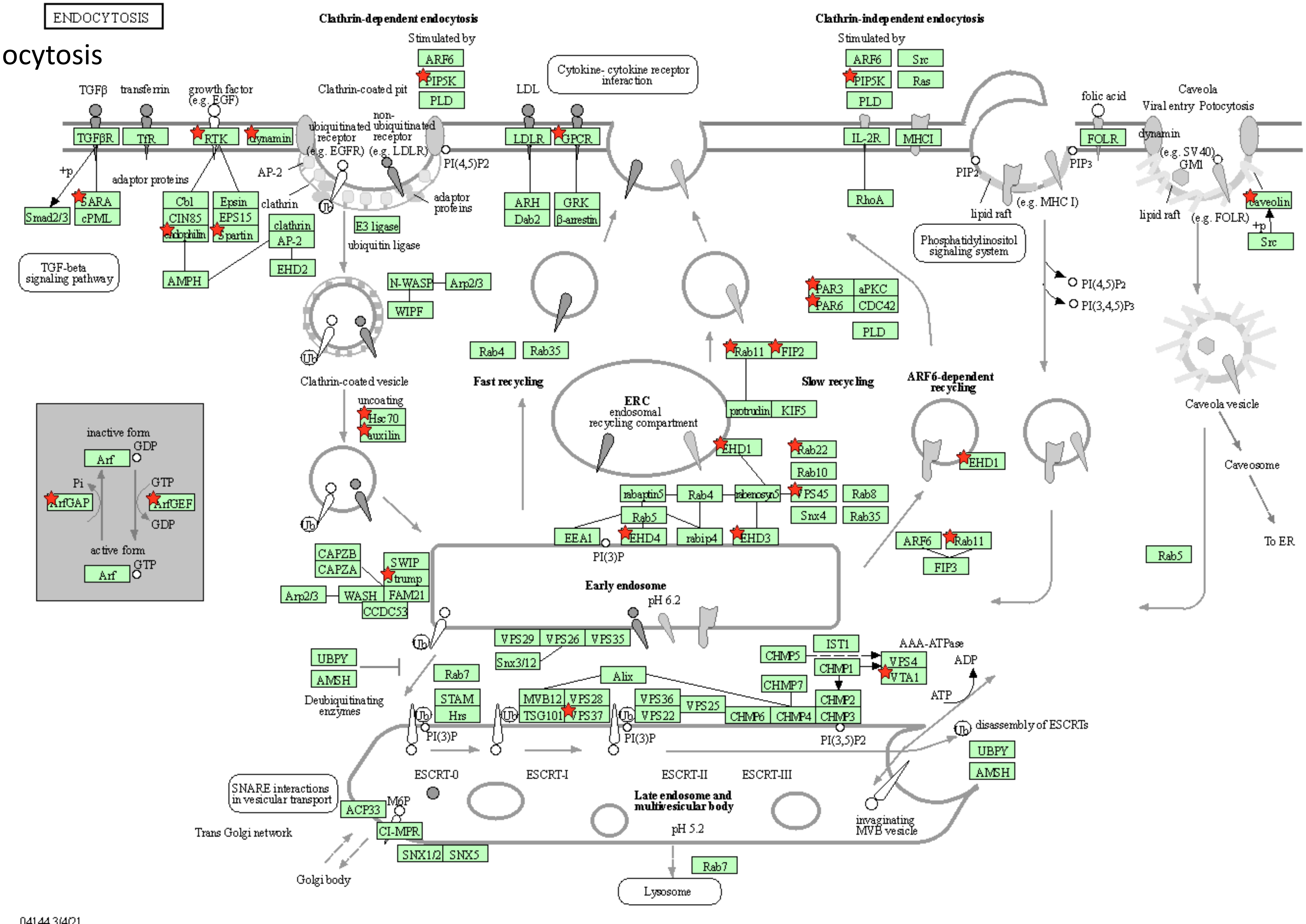

Long-term potentiation

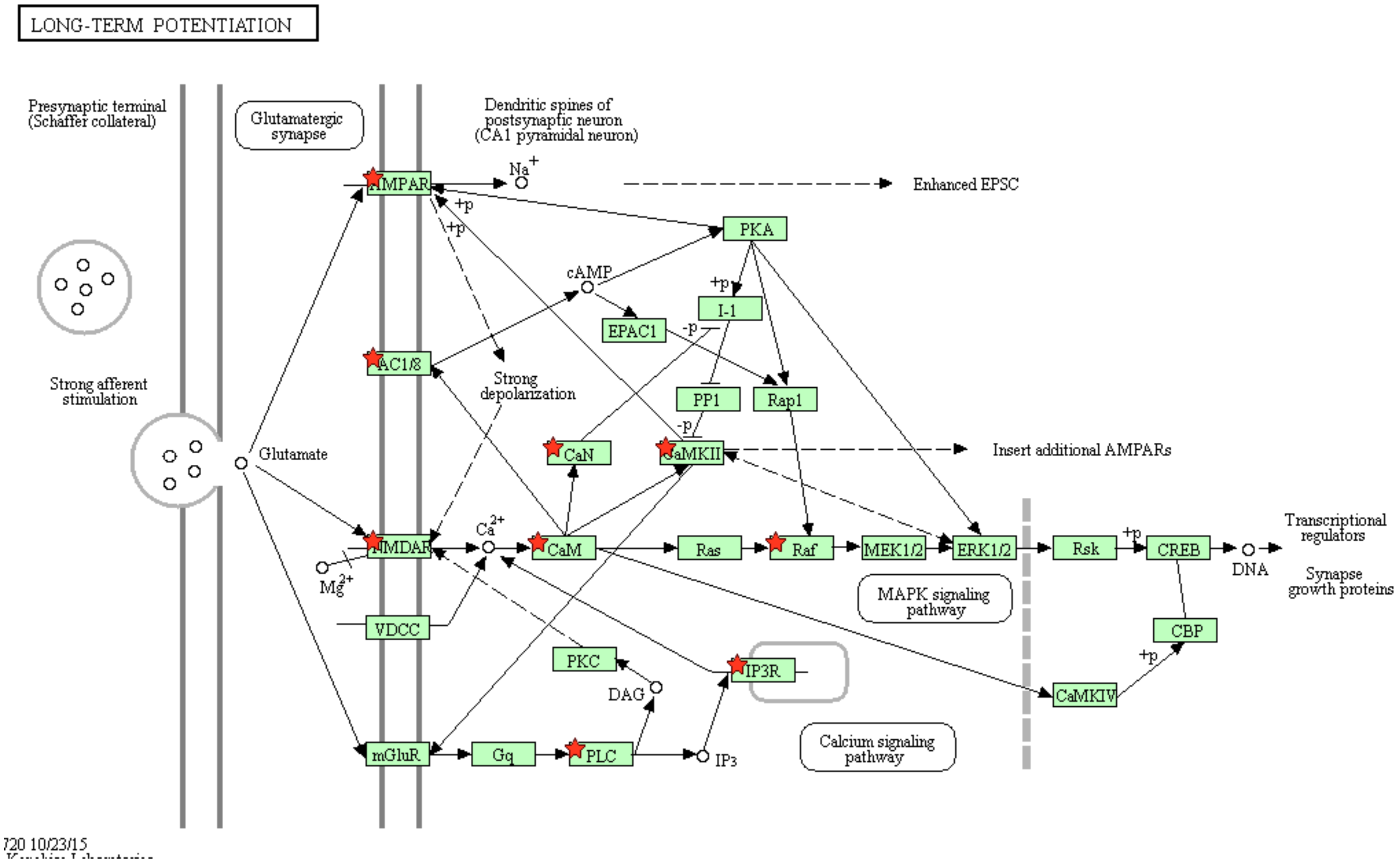

Axon guidance

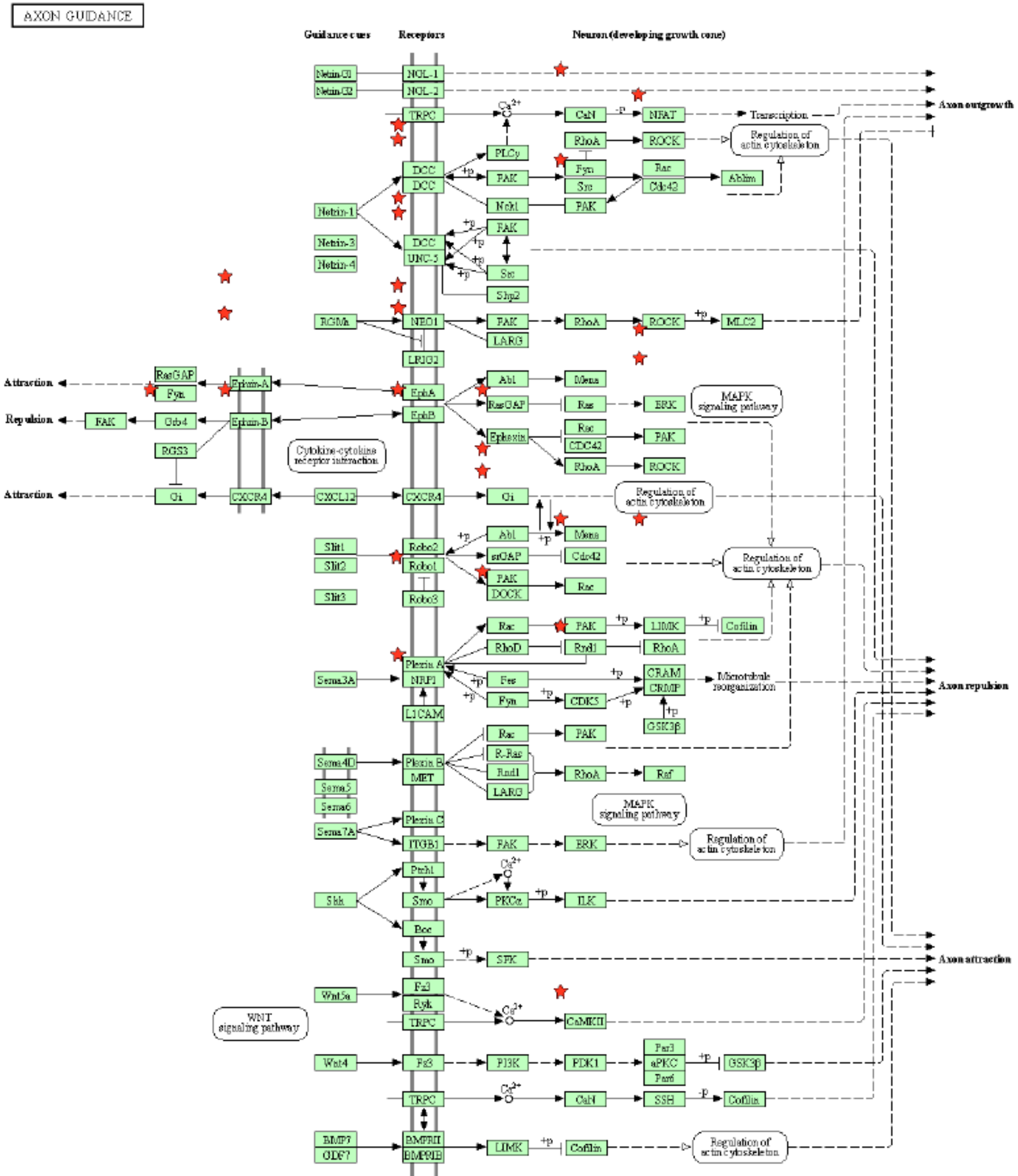

Serotonergic synapse

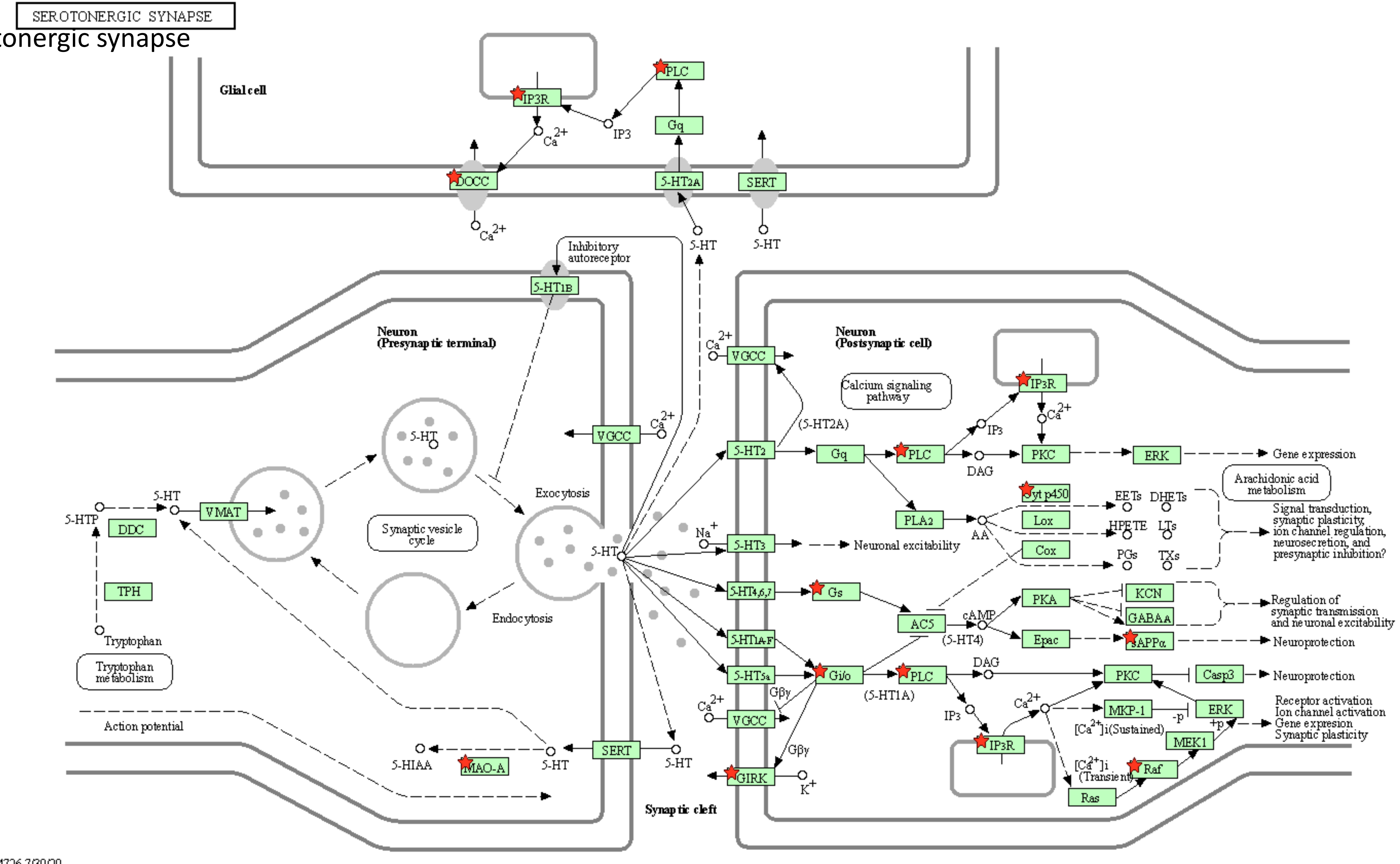

Supplement: Supplementary file 6 — Additional file 6. KEGG pathway analysis used the data in Yoshizawa et al., 2018. [file 12915_2023_1725_MOESM6_ESM.pdf]
